# Prevalence and predictors of oral rehydration therapy, zinc, and other treatments for diarrhoea among children under-five in sub-Saharan Africa

**DOI:** 10.1371/journal.pone.0275495

**Published:** 2022-10-13

**Authors:** Bright Opoku Ahinkorah, Richard Gyan Aboagye, Abdul-Aziz Seidu, James Boadu Frimpong, Abdul Cadri, Agani Afaya, John Elvis Hagan, Sanni Yaya

**Affiliations:** 1 REMS Consult Limited, Sekondi Takoradi, Western Region, Ghana; 2 School of Public Health, Faculty of Health, University of Technology Sydney, Sydney, Australia; 3 Department of Family and Community Health, Fred N. Binka School of Public Health, University of Health and Allied Sciences, Hohoe, Ghana; 4 College of Public Health, Medical and Veterinary Sciences, James Cook University, Townsville, Australia; 5 Centre for Gender and Advocacy, Takoradi Technical University, Takoradi, Ghana; 6 Department of Health, Physical Education, and Recreation, University of Cape Coast, Cape Coast, Ghana; 7 Department of Social and Behavioural Science, School of Public Health, University of Ghana, Legon- Accra, Ghana; 8 Department of Family Medicine, Faculty of Medicine, McGill University, Montreal, Quebec, Canada; 9 Mo-Im Kim Nursing Research Institute, Yonsei University, College of Nursing, Seoul, South Korea; 10 Department of Nursing, School of Nursing and Midwifery, University of Health and Allied Sciences, Ho, Ghana; 11 Neurocognition and Action-Biomechanics-Research Group, Faculty of Psychology and Sport Sciences, Bielefeld University, Bielefeld, Germany; 12 School of International Development and Global Studies, University of Ottawa, Ottawa, Canada; 13 The George Institute for Global Health, Imperial College London, London, United Kingdom; University Hospital Heidelberg, GERMANY

## Abstract

**Background:**

Despite the evidence-based effectiveness of diarrhoea treatment in preventing diarrhoea-related child mortality, the accessibility and utilization of diarrhoea treatments remain low in sub-Saharan Africa, even though these treatments are available. Therefore, this study aimed to assess the prevalence and predictors of diarrhoea treatment among under-five children in sub-Saharan Africa.

**Methods:**

This study involved cross-sectional analyses of secondary data from the most recent Demographic and Health Surveys of 30 countries in sub-Saharan Africa. Percentages with their respective 95% confidence intervals (CI) were used to summarise the prevalence of diarrhoea treatment. A multivariable multilevel binary logistic regression analysis was employed to examine the predictors of diarrhoea treatment among children under five years in sub-Saharan Africa. The regression results were presented using adjusted odds ratio with their accompanying 95% confidence intervals. Statistical significance was set at p<0.05. Stata software version 16.0 was used for the analyses.

**Results:**

The overall prevalence of diarrhoea treatment among under-five children in sub-Saharan Africa was 49.07% (95% CI = 44.50–53.64). The prevalence of diarrhoea treatment ranged from 23.93% (95% CI = 20.92–26.94) in Zimbabwe to 66.32% (95% CI = 61.67–70.97) in Liberia. Children aged 1 to 4 years, those whose mothers had at least primary education, those whose mothers had postnatal care visits, those whose mothers believed that permission to go and get medical help for self was a big problem, and those whose mothers’ partners had at least primary education were more likely to undergo diarrhoea treatment as compared to their counterparts. The odds of diarrhoea treatment increased with increasing wealth index with the highest odds among those in the richest quintile. Also, the odds of diarrhoea treatment was higher in the Central, Eastern, and Western geographical subregions compared to those in the Southern geographical subregion. However, children whose mothers were cohabiting, those whose mothers were exposed to watching television, and those living in female-headed households were less likely to undergo diarrhoea treatment.

**Conclusion:**

The study found that the prevalence of diarrhoea treatment among children in sub-Saharan Africa was relatively low and varied across countries. The sub-regional estimates of diarrhoea treatment and identified associated factors can support country-specific needs assessments targeted at improving policy makers’ understanding of within-country disparities in diarrhoea treatment. Planned interventions (e.g., provision of quality and affordable supply of oral rehydration salts and zinc) should seek to scale up diarrhoea treatment uptake among under-five children in sub-Saharan Africa with much focus on the factors identified in this study.

## Introduction

Diarrhoea disease is defined as the loss of stool consistency, with pasty or liquid stools, and/ or an increase in stool frequency to more than three stools in 24 hours with or without fever, or vomiting [1]. Deaths due to diarrhoea are usually preventable; unfortunately, the burden remains high [2]. The highest burden of diarrhoea is observed in low-and middle-income countries [3]. The Global Burden of Diseases, Injuries, and Risk factors study, 2016 [4] estimated that diarrhoea was the third leading cause of death among children under five in 2015, responsible for an estimated 330,000 deaths and 30 million severe cases worldwide. Persistent diarrhoea leads to undernutrition [5] and profoundly impairs the growth and development of children under the age of five [6]. It has been reported that diarrhoea is associated with micronutrient deficiencies, impaired neurodevelopment, and increased morbidity and mortality from other childhood diseases [5, 6].

The management of diarrhoea is very important for children in sub-Saharan African countries. As a result, several interventions have been implemented including effective treatment modalities such as oral rehydration therapy, zinc treatment, continued feeding, and antibiotic treatment for certain bacterial diarrhoea, noting that almost all deaths due to diarrhoea could be prevented if these treatment modalities are highly accessed [2, 3]. Zinc and oral rehydration therapy are known to have significant roles in childrens’ ability to recover from diarrhoea [3, 7]. Despite the evidence-based effectiveness of diarrhoea treatment in preventing diarrhoea-related child mortality, the accessibility of diarrhoea treatments remains low in sub-Saharan Africa (SSA), even though these treatments are available [7–9]. Ugwu et al. [9] reported that the prevalence of zinc treatment and oral rehydration therapy (ORT) for diarrhoea were 6% and 21%, respectively in Nigeria. Another study in East Africa [10] reported the prevalence of diarrhoea treatment to be 15.1% in Burundi, 8.2% in Kenya, 20.1% in Zimbabwe, 28.4% in Malawi, and 40.5% in Uganda.

Several factors are associated with accessing treatments for diarrhoea among children in SSA. A study by Kawakatsu, et al. [11] in Kenya reported that household wealth and severity of diarrhoea were significantly associated with accessing diarrhoea treatment. It was indicated that children who belonged to the middle wealth quintile had twice the odds of receiving treatment for diarrhoea compared to children of the lower wealth quintile. Also, childhood diarrhoea with blood presented an increased likelihood of accessing treatment, compared to childhood diarrhoea without blood. The type of facility a child is taken to is also reported to be associated with diarrhoea treatment, as Sood and Wagner [12] found that children who receive treatment from private facilities are less likely to be given ORT for diarrhoea. Another study in Uganda [13] indicated that price and convenience of service are key predictors of diarrhoea treatment accessibility. In that study, it was further stated that free access to diarrhoea treatment significantly increased the likelihood of accessing treatment for diarrhoea. Also, households’ convenience to health facilities increased odds of accessing treatment for diarrhoea. Another study in East Africa reported that high maternal education and high community media exposure were significantly associated with a higher prevalence of diarrhoea treatment utilization [10]. Other studies have found knowledge about treatment and area of residence as the factors that are significantly associated with accessing diarrhoea treatment [14–17].

Given that diarrhoea-related deaths among children in SSA can be prevented, it is important to scale up diarrhoea interventions, including treatment in SSA. Despite the many advantages of diarrhoea treatment among children, treatment prevalence is low in SSA [9, 10]. There have been studies in SSA that have been on diarrhoea treatment and associated factors; however, these studies are mostly country level and use different statistical methods, making it difficult to generalize findings at the sub-Saharan African regional level. Therefore, this study was conducted to fill the gap in literature accordingly. As this study is the first to assess the prevalence and predictors of diarrhoea treatment at the sub-Saharan African regional level, findings can provide a basis for scaling up diarrhoea treatment in SSA.

## Materials and methods

### Data source and study design

Data for the study were pooled from the most recent Demographic and Health Surveys (DHS) of thirty countries in SSA published from 2010 to 2020. The study included countries whose datasets had information on diarrhoea treatment and the other variables considered in this study. Specifically, we pooled the data from the child’s recode file (KR File). The DHS provides a comparably-representative dataset on health and social indicators such as child and maternal health of which diarrhoea treatment is a component, in over 85 low-and-middle countries where the survey is conducted periodically [18]. DHS employed a cross-sectional study design to collect data from the respondents. The respondents were sampled using a two-stage cluster sampling technique. The first stage of sampling in the DHS consisted of compiling a list of primary sampling units (PSUs) or enumeration areas (EAs) that covered the entire country and were obtained from the most recent national census. The EAs were then divided into standardized segments. Then, with a probability proportional to the size of the EA, a random sample of a predetermined segment is chosen. In the second stage, households were systematically selected from a list of previously enumerated households in each selected EA segment, and those who were regular residents of the selected households or visitors who slept in the households the night before the survey were interviewed. Structured questionnaires were used to collect data from the respondents. The total number of children 30,217 was included in the final analyses. This sample consisted of only those with complete observations of the variables of interest (Table 1). The dataset used in our study is freely available at https://dhsprogram.com/data/available-datasets.cfm.

**Table 1:** Description of the study sample.

Countries	Year of survey	Total number of children	Unweighted	Weighted N	Weighted %
			N		
1. Angola	2015–16	14322	960	1,058	3.5
2. Burkina Faso	2010	15044	1572	1,588	5.3
3. Benin	2017–18	13589	980	963	3.2
4. Burundi	2016–17	13192	1845	1,982	6.6
5. Congo DR	2013–14	18716	1698	1,701	5.6
6. Congo	2011–12	9329	887	849	2.8
7. Cote d’Ivoire	2011–12	7776	786	761	2.5
8. Cameroon	2018	9733	657	678	2.2
9. Ethiopia	2016	10641	838	955	3.2
10. Gabon	2012	6067	435	370	1.2
11. Ghana	2014	5884	452	397	1.3
12. Gambia	2019–20	8386	1099	937	3.1
13. Guinea	2018	7951	753	773	2.6
14. Kenya	2014	20964	957	928	3.1
15. Comoros	2012	3149	258	273	0.9
16. Liberia	2019–20	5704	511	396	1.3
17. Lesotho	2014	3138	246	254	0.8
18. Mali	2018	9940	10655	1,099	3.6
19. Malawi	2015–16	17286	2478	2,569	8.5
20. Nigeria	2018	33924	2855	2798	9.3
21. Niger	2012	12558	1158	1268	4.2
22. Namibia	2013	5046	275	266	0.9
23. Sierra Leone	2019	9899	376	386	1.3
24. Senegal	2010–11	12326	1315	1,292	4.3
25. Chad	2014–15	18623	699	754	2.5
26. Togo	2013–14	6979	732	664	2.2
27. Tanzania	2015–16	10233	786	771	2.5
28. Uganda	2016	15522	1920	1,820	6.0
29. Zambia	2018	9959	904	897	3.0
30. Zimbabwe	2015	6132	720	770	2.5
**All countries**	**2010–2020**	**341988**	**30217**	**30217**	**100.0**

### Study variables

#### Outcome variable

The outcome variable was diarrhoea treatment. With this variable, only mothers whose children had reported having diarrhoea two weeks prior to the survey were considered. The treatments were fluid from an Oral Rehydration Salts (ORS) packet or pre-packaged ORS fluid, recommended homemade fluids (RHF), ORS or RHF, Zinc, ORS and zinc, ORS or increased fluids, oral rehydration therapy (ORT), continued feeding and ORT, and other treatments (not antibiotic, antimotility, zinc). Those whose response option was “don’t know” were excluded from the study. Further, we coded the remaining responses into “1 = Had diarrhoea treatment” and “0 = No diarrhoea treatment”.

### Explanatory variables

The explanatory variables included in the study were sectioned into individual level (consisting of child and mother characteristics) and household/community level variables. The variables were nineteen in all. Variables considered at the individual level included sex of child, age of child, birth order, size of child at birth, type of delivery, twin status, age of the mother, educational level of the mother and the partner/husband, marital status, current working status, antenatal care attendance, place of delivery, postnatal care attendance, health insurance coverage, person who decides on respondents health care, getting medical help for self: getting money for treatment, getting medical help for self: distance to health facility, getting medical help for self: permission to go, exposure to radio, exposure to television, and exposure to reading newspaper or magazine. Household size, wealth index, place of residence, and geographical subregions were the household/ community level variables in the study. For the variables (getting medical help for self: getting money for treatment, getting medical help for self: distance to health facility, and getting medical help for self: permission to go to the health facility), the women were asked to respond to questions concerning the barriers to accessing healthcare based on money, distance, and permission to ascertain whether it’s a problem for them or not. The response options were “not a big problem” and “a big problem”. The choice of the explanatory variables was influenced by literature [10, 12] as well as their availability in the DHS dataset.

### Statistical analyses

Stata software version 16.0 (Stata Corporation, College Station, TX, USA) was used for the analysis. Forest plot was used to summarise the prevalence of diarrhoea treatment among the children in SSA. The forest plot was generated using the ‘metan’ command in stata. First, we calculated the standard error for each prevalence and the random-effects which produces the prevalence, 95% confidence intervals and effect sizes (weight). Cross-tabulation was adopted to examine the distribution of the diarrhoea treatment across the explanatory variables. The variables significantly associated with diarrhoea treatment use were determined using the Pearson chi-square test of independence. The best selection method command in Stata “gvselect” was used to select the best-fitted set of variables for the multilevel binary logistic regression. Four multilevel logistic regression models were built to determine the predictors of diarrhoea treatment. The first model (Model O) was fitted to show the variation in the diarrhoea treatment caused by the clustering of the primary sampling units (PSUs) and the explanatory variables. Model I included individual-level variables against diarrhoea treatment. Model II included the household/community level variables. Model III was fitted with all explanatory variables versus diarrhoea treatment. We used Akaike’s Information Criterion (AIC) to evaluate model fitness and model comparison. We applied weighting in all the analyses. First, we weighted the dataset for each country using the commands (gen wt = v005/1000000) to obtain unbiased estimates according to the DHS guidelines. Additionally, the survey command in Stata (svy [svyset v021 [pweight = wt], strata(v023)]) was used to adjust for the complex sampling structure of the data in all the analysis. Afterwards, the dataset for the countries were appended together and used for the analysis. The result of the regression analysis was presented using an adjusted odds ratio (aOR), with their 95% confidence intervals (CIs). Statistical significance was set at p<0.05. We adhered to the guidelines outlined in the Strengthening the Reporting of Observational Studies in Epidemiology (STROBE) in drafting this paper [19].

### Ethical consideration

Due to the public availability of the DHS dataset, ethical approval was not sought. However, permission to use the dataset was obtained from the MEASURE DHS. All ethical standards and guidelines concerning the use of the DHS dataset for publication were adhered to. Information on the ethical standards can be found at http://goo.gl/ny8T6X.

## Results

### Prevalence of diarrhoea treatment in sub-Saharan Africa

**Fig 1:**
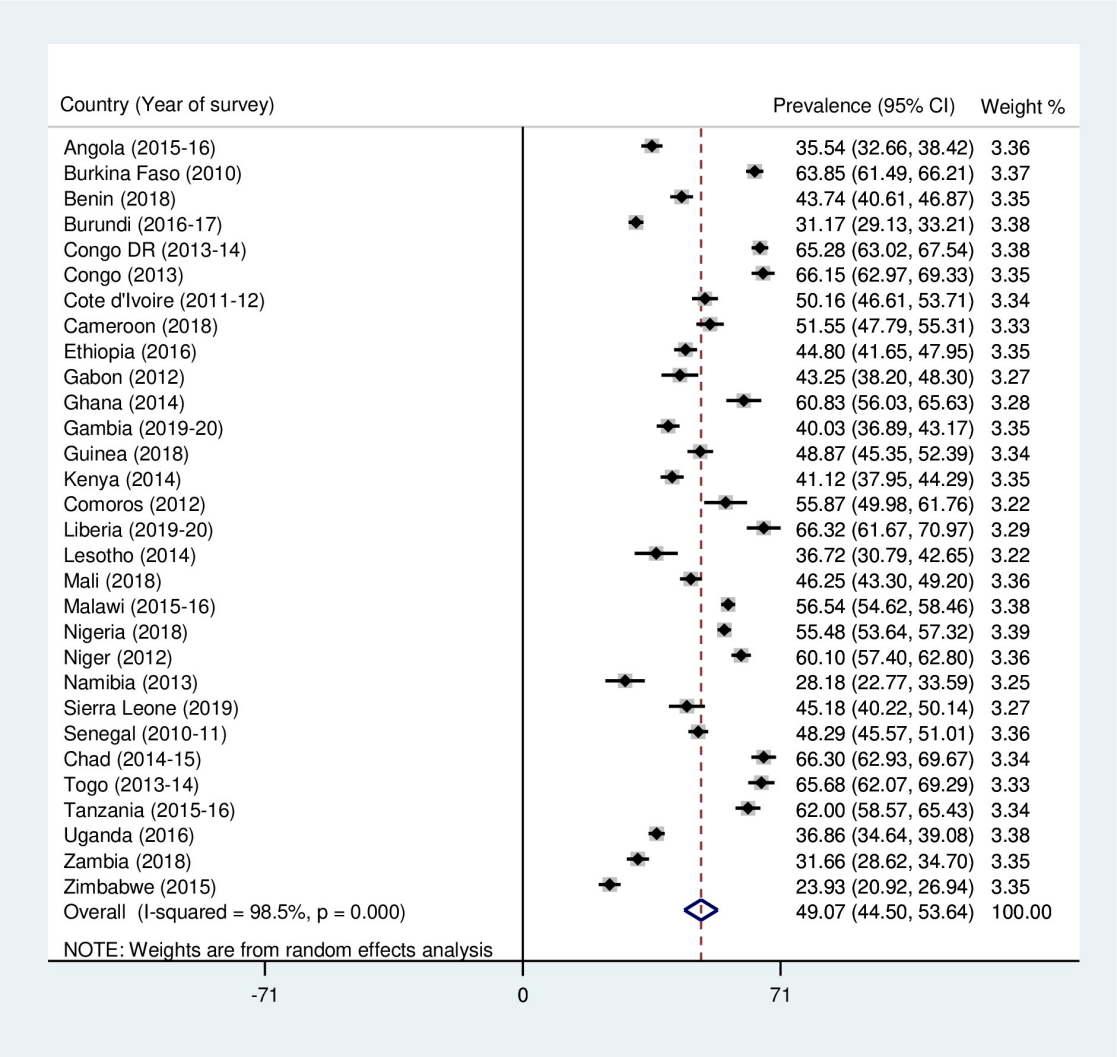
Forest plot showing the prevalence of oral rehydration therapy, zinc, and other treatments for diarrhoea among children under five in sub-Saharan Africa.

Fig 1 shows the prevalence of diarrhoea treatment among children under five in SSA. The study found that the prevalence of diarrhoea treatment among children in SSA was 49.07% (95% CI = 44.50–53.64). The prevalence of diarrhoea treatment ranged from 23.93% (95% CI = 20.92–26.94) in Zimbabwe to 66.32% (95% CI = 61.67–70.97) in Liberia.

### Chi-square test showing the distribution of diarrhoea treatment across explanatory variables

Table 2 outlines the results of the Chi-square test showing differences in diarrhoea treatment across the explanatory variables. The study found that age of child (*p* < 0.001), size of child at birth (*p* = 0.001), marital status (*p* = 0.010), current working status (*p* = 0.001), postnatal care (PNC) attendance (*p* < 0.001), person who usually decides on respondent’s health care (*p* < 0.001), getting medical help for self: permission to go to the health facility (*p* < 0.001), getting medical help for self: getting money for treatment (*p* < 0.001), exposure to watching television (*p* = 0.022), exposure to listening to radio (*p* = 0.014), head of household (*p* < 0.001), and wealth index (*p* = 0.001) were significantly associated with diarrhoea treatment.

**Table 2:** Distribution of diarrhoea treatment across the explanatory variables.

Variables	Weighted N	Weighted %	Diarhea treatment		
			No (%)	Yes (%)	p-value
**Sex of child**					0.072
Male	15,837	52.4	49.7	50.3	
Female	14,380	47.6	51.0	49.0	
**Age of child**					<0.001
0	9,348	30.9	55.1	44.9	
1	11,586	38.3	48.5	51.5	
2	5,819	19.3	47.1	52.9	
3	2,365	7.8	48.3	51.7	
4	1,098	3.7	51.0	49.0	
**Birth order**					0.134
1	5,541	18.3	50.4	49.6	
2	5,704	18.9	51.4	48.6	
3	9,972	16.4	51.7	48.3	
4	4,041	13.4	49.8	50.2	
5 and above	9,9058	33.0	49.3	50.7	
**Size of child at birth**					0.001
Very large	3,808	12.6	47.5	52.5	
Large	7,322	24.2	49.2	50.8	
Average	13,648	45.2	51.6	48.4	
Small than average	3,683	12.2	49.8	50.2	
Very small	1,761	5.8	52.3	47.7	
**Type of delivery**					0.346
Vaginal	28873	95.6	50.3	49.7	
Caesarean section	1344	4.4	52.1	47.9	
**Child is a twin**					0.162
Single birth	29699	98.3	50.4	49.6	
Multiple birth	518	1.7	46.6	53.4	
**Mother’s age**					0.386
15–19	1212	7.0	51.2	48.8	
20–24	7602	25.2	51.1	48.9	
25–29	8273	27.4	50.1	49.9	
30–34	5973	19.8	50.4	49.6	
35–39	3961	13.1	48.5	51.5	
40–44	1779	5.9	50.4	49.6	
45–49	500	1.7	53.4	46.6	
**Maternal educational level**					0.636
No formal education	12434	41.2	50.1	49.9	
Primary	10317	34.1	50.8	49.2	
Secondary or higher	7467	24.7	50.1	49.9	
**Marital status**					0.010
Married	24031	79.5	49.8	50.2	
Cohabiting	6186	20.5	52.4	47.6	
**Current working status**					0.001
Not working	10697	35.4	52.0	48.0	
Working	19520	64.6	49.4	50.6	
**Antenatal care attendance**					0.111
None	2711	9.0	51.6	48.4	
1–3	10903	36.1	49.3	50.7	
4 or more	16603	54.9	50.8	49.2	
**Place of delivery**					0.064
Home	9480	31.4	50.0	50.0	
Health facility	20329	67.3	50.4	49.6	
Other	408	1.3	57.4	42.6	
**Postnatal care attendance**					<0.001
No	17318	57.3	51.7	48.3	
Yes	12899	42.7	48.5	51.5	
**Health insurance coverage**					0.465
No	28629	94.7	50.3	49.7	
Yes	1588	4.3	51.5	48.5	
**Person who usually decides on respondent’s health care**					<0.001
Partner alone/Someone else/others	15184	50.2	47.7	52.3	
Respondent alone	4824	16.0	53.6	46.4	
Respondent and partner	10209	33.8	52.7	47.3	
**Getting medical help for self: permission to go** to the health facility					<0.001
Not a big problem	24095	79.7	51.3	48.7	
Big problem	6122	20.3	46.4	53.6	
**Getting medical help for self: distance to health facility**					0.478
Not a big problem	17289	57.2	50.6	49.4	
Big problem	12928	42.8	50.0	50.0	
**Getting medical help for self: getting money for treatment**					<0.001
Not a big problem	13022	43.1	51.9	48.1	
Big problem	17195	56.9	49.2	50.8	
**Exposure to watching television**					0.022
No	22976	76.0	49.8	50.2	
Yes	7241	24.0	52.0	48.0	
**Exposure to listening to radio**					0.014
No	19101	63.2	49.7	50.3	
Yes	11116	36.8	51.5	48.5	
**Exposure to reading newspaper/magazine**					0.209
No	28597	94.6	50.2	49.8	
Yes	1620	5.4	52.4	47.6	
**Partner’s educational level**					0.272
No formal education	10570	35.0	50.2	49.8	
Primary	8932	29.6	51.2	48.8	
Secondary or higher	10715	35.4	49.8	50.2	
**Household size**					0.099
Small	13227	43.8	51.1	48.9	
Medium	12709	42.0	50.0	50.0	
Large	4281	14.2	48.9	51.1	
**Head of household**					<0.001
Male	26061	86.2	49.8	50.2	
Female	4156	13.8	53.6	46.4	
**Wealth index**					0.001
Poorest	6979	23.1	52.7	47.3	
Poorer	6845	22.7	50.9	49.1	
Middle	5961	19.7	49.8	50.2	
Richer	5927	19.6	49.3	50.7	
Richest	4505	14.9	47.9	52.1	
**Place of residence**					0.363
Urban	9050	30.0	49.7	50.3	
Rural	21167	70.0	50.6	49.4	

*p-values were obtained from chi-square test.

### Predictors of diarrhoea treatment among children in sub-Saharan Africa

#### Fixed effect results

Table 3, Model III outlines the predictors of diarrhoea treatment among children in SSA. Compared to children aged below 1 year, those aged 1 to 4 years had higher odds of receiving diarrhoea treatment. The odds of diarhoea treatment increases with increasing maternal educational level, with the highest odds among women with secondary or higher level [aOR = 1.19, 95% CI = 1.07, 1.32]. Children whose mothers attended postnatal care [aOR = 1.21, 95% CI = 1.13, 1.29] and mothers who believed that permission to go for medical care is a problem [aOR = 1.12, 95% CI = 1.03, 1.22] had higher odds of diarrhoea treatment. Relative to partner’s with no formal education, those with at least primary education were more likely to seek diarrhoea treatment. The odds of diarrhoea treatment increased with wealth index with the highest odds in those with the highest wealth quintile [aOR = 1.33, 95% CI = 1.18, 1.50]. Children from Central [aOR = 3.81, 95% CI = 3.24, 4.48], Eastern [aOR = 2.16, 95% CI = 1.88, 2.48], and Western Africa [aOR = 3.45, 95% CI = 2.97, 3.99] were more likely to receive diarrhoea treatment compared to those from the Southern Africa. However, children whose mothers were cohabiting, those whose mothers were exposed to watching television, and those who lived in female-headed households were less likely to undergo diarrhoea treatment compared to their counterparts.

**Table 3:** Fixed and random effect analysis of predictors of diarrhoea treatment among children in sub-Saharan Africa.

Variable	Model O	Model I	Model II	Model III
		aOR [95% CI]	aOR [95% CI]	aOR [95% CI]
**Fixed effect results**				
**Age of child**				
0		1.00		1.00
1		1.32*** [1.24, 1.41]		1.33*** [1.24, 1.42]
2		1.38*** [1.27, 1.49]		1.38*** [1.28, 1.50]
3		1.31*** [1.16, 1.48]		1.33*** [1.18, 1.50]
4		1.19*[1.01, 1.39]		1.22* [1.04, 1.44]
**Maternal educational level**				
No formal education		1.00		1.00
Primary		1.01 [0.93, 1.09]		1.16*** [1.07, 1.26]
Secondary or higher		1.06 [0.96, 1.17]		1.19** [1.07, 1.32]
**Marital status**				
Married		1.00		1.00
Cohabiting		0.90*[0.83, 0.98]		0.81*** [0.74, 0.88]
**Current working status**				
Not working		1.00		1.00
Working		1.11*** [1.05, 1.18]		1.02 [0.96, 1.08]
**Place of delivery**				
Home		1.00		1.00
Health facility		1.01 [0.94, 1.08]		1.05 [0.98, 1.13]
Other		0.79* [0.62, 1.00]		0.89 [0.70, 1.14]
**Postnatal care attendance**				
No		1.00		1.00
Yes		1.16*** [1.09, 1.24]		1.21*** [1.13, 1.29]
**Person who usually decides on respondent’s health care**				
Respondent alone		1.00		1.00
Respondent and partner		1.06 [0.97, 1.15]		0.95 [0.87, 1.04]
Partner alone/Someone else/others		1.31*** [1.20, 1.42]		1.03 [0.94, 1.14]
**Getting medical help for self: permission to go**				
Not a big problem		1.00		1.00
Big problem		1.21*** [1.12, 1.32]		1.12** [1.03, 1.22]
**Getting medical help for self: getting money for treatment**				
Not a big problem		1.00		1.00
Big problem		1.07* [1.00, 1.15]		1.02 [0.95, 1.09]
**Getting medical help for self: distance to health facility**				
Not a big problem		1.00		1.00
Big problem		0.94 [0.88, 1.00]		1.00 [0.93, 1.06]
**Partner’s educational level**				
No formal education		1.00		1.00
Primary		1.03 [0.95, 1.12]		1.18*** [1.09, 1.29]
Secondary or higher		1.10* [1.00, 1.20]		1.11*[1.02, 1.22]
**Exposure to watching television**				
No		1.00		1.00
Yes		0.89** [0.82, 0.97]		0.72*** [0.66, 0.79]
**Exposure to listening to radio**				
No		1.00		1.00
Yes		0.94 [0.88, 1.00]		0.95 [0.89, 1.01]
**Household size**				
Small			1.00	1.00
Medium			0.99 [0.93, 1.06]	1.01 [0.95, 1.08]
Large			0.92 [0.84, 1.02]	0.95 [0.87, 1.05]
**Head of household**				
Male			1.00	1.00
Female			0.90*[0.82, 0.99]	0.91*[0.83, 0.99]
**Wealth index**				
Poorest			1.00	1.00
Poorer			1.06 [0.97, 1.15]	1.05 [0.96, 1.14]
Middle			1.11*[1.02, 1.21]	1.12** [1.03, 1.23]
Richer			1.14** [1.03, 1.25]	1.16** [1.05, 1.29]
Richest			1.21*** [1.10, 1.34]	1.33***[1.18, 1.50]
**Geographical subregions**				
Southern Africa			1.00	1.00
Central Africa			3.16*** [2.72, 3.68]	3.81*** [3.24, 4.48]
Eastern Africa			1.96*** [1.72, 2.23]	2.16*** [1.88, 2.48]
Western Africa			2.88*** [2.51, 3.30]	3.45*** [2.97, 3.99]
**Random effect results**				
PSU variance (95% CI)	0.09 [0.07, 0.12]	0.09 [0.07, 0.12]	0.09 [0.07, 0.12]	0.09 [0.07, 0.12]
ICC	0.0279065	0.0281329	0.0276164	0.0269776
Wald chi-square	Reference	260.44***	333.33***	610.45***
**Model fitness**				
Log-likelihood	-21028.437	-20839.611	-20686.268	-20478.82
AIC	42060.87	41723.22	41396.54	41021.64
N	30217	30217	30217	30217
Number of clusters	1326	1326	1326	1326

aOR = adjusted odds ratios; CI Confidence Interval

* *p* < 0.05

** *p* < 0.01

*** *p* < 0.001

1.00 = Reference category; PSU = Primary Sampling Unit; ICC = Intra-Class Correlation; AIC = Akaike’s Information Criterion; N = Sample size.

### Random effect results

Results from Table 3, Model O showed that diarrhoea treatment varies significantly across the clusters (σ 2 = 0.09, 95% CI = 0.07, 0.12). Also, Model O showed that the between-cluster variations accounted for 2.8% of the diarrhoea treatment (ICC = 0.0279065). The between-cluster variation approximately remained the same for Model O, I, and II. However, it reduced to 2.7% in the model containing all the explanatory variables (individual and contextual-level variables) [Model III] (ICC = 0.0269776). This shows that the variations in the probability of a child receiving diarrhoea treatment varies across the clusters. Additionally, AIC decreased from Model O to Model III. Hence, Model III was chosen as the best fitted-model for the study.

## Discussion

The study assessed the prevalence and predictors of diarrhoea treatment among children in SSA. The study found the prevalence of diarrhoea treatment among children in SSA to be 49.07%. The low prevalence of diarrhoea treatment observed in this study is similar to a recent study conducted among LMICs which reported low rates of ORT coverage in some central sub-Saharan Africa countries (Cameroon, 24.2%; Gabon, 32.8%), and parts of western (Ghana, 39.8%; Nigeria, 40.8%) and eastern SSA (Ethiopia, 28.1%) [20]. Previous studies have attributed this low coverage rate to doctor and patient knowledge about ORT, ORS supply, cost, and taste; and access to clean water [21, 22]. Empirical evidence have shown that improvements in ORT coverage can be driven by changes in governmental policies, media campaigns, and community culture and beliefs concerning diarrhoeal treatment [20, 23, 24]. The prevalence of diarrhoea treatment ranged from 23.93% in Zimbabwe to 66.32% in Liberia. A plausible reason for this finding in the case of Liberia could be attributed to the implementation of the Community Health Worker (CHW) program which significantly led to the improvement in children’s rate of receiving paediatric treatment from a qualified health provider, especially in the remote areas [25].

The study found that children who were aged 1 year and above were more likely to undergo diarrhoea treatment compared to children who were aged below 1. A possible reason for this finding could be that one main way of suspecting diarrhoea at the household level is the passage of watery stool; since babies aged below 1 mostly pass watery stools due to the foods they eat, symptoms of diarrhoea may be missed and treatment not sought compared to children aged 1 year and above whose stools are not expected to be watery due to the food eaten. Hence any watery stool can be an indication of diarrhoea and treatment sought [26, 27]. Also, children aged one year and above can express any discomfort to parents and seek medical treatment compared to those aged 0 who might not be able to express discomfort well [28, 29].

Children whose mothers had attained at least primary education were more likely to seek diarrhoea treatment for their children than those whose mothers had no formal education. Educated women could have been more knowledgeable about the diverse diarrhoea treatment options, increasing their likelihood to access diarrhoea treatment services for their children experiencing diarrhoea [30]. This findings could imply that the education acquired could have also increased their understanding and knowledge on the importance of early health care seeking, thus, early diarrhoea treatment.

Children whose mothers had postnatal care visits were more likely to undergo diarrhoea treatment than those whose mothers did not have postnatal care visits. Women who had frequent postnatal care visitations could have been educated on the importance of timely diarrhoea treatment, resulting in their increased likelihood of treating their childrens’ diarrhoea [31, 32]. The study also found that children whose mothers believed that permission to go and get medical help for self was a big problem were more likely to access diarrhoea treatment compared with those whose mothers believed that permission to go and get medical for self was not a big problem. The plausible explanation for this is that the permission to go and get medical treatment could be a big problem for self (mothers themselves) but not for children; hence, they got permission to seek treatment for children.

Children whose mothers’ partners had attained at least primary education were more likely to undergo diarrhoea treatment than those whose mothers’ partners had no formal education. Educated male spouses may have been well informed about the importance of accessing timely diarrhoea treatment services for their children suffering from diarrhoea disease and are more likely to convince their partners to undergo diarrhoea treatment in such instances [33, 34].

Similar to the findings of other previous studies [11, 26, 35], this study found that children whose mothers’ were wealthy were more likely to undergo diarrhoea treatment compared with those whose mothers were not. A possible reason for this finding could be that wealthy women have the financial capacity to afford diarrhoea treatment options such as zinc treatment, ORT, and antibiotics, increasing their likelihood to access diarrhoea treatment [11, 36]. Aside from paying the health services cost, wealthy women are more likely to afford transportation costs as they seek health services [36].

However, children whose mothers were cohabiting were less likely to undergo diarrhoea treatment compared to those whose mothers were married. An acceptable reason for this finding could be that women who are cohabiting are not financially empowered, reducing their likelihood to access appropriate diarrhoea treatment modalities including zinc tablets, ORT, and antibiotics [37]. Moreover, this study has noted that children whose mothers made healthcare decisions with their partners were less likely to undergo diarrhoea treatment compared with those whose mothers’ healthcare decision was determined by someone else or others. This could plausibly be due to the effect of gender roles and relations which give men the power to make decisions in the family; hence, if the man decides that treatment should not be sought for diarrhoea, women mostly are obliged to abide by it [38].

The positive influence of mass media exposure on maternal and child health-seeking behaviors has been well documented in the literature [39–42]. However, our study finding is counter-intuitive with the above-mentioned evidence from previous studies. We found that children whose mothers were exposed to watching television were less likely to undergo diarrhoea treatment compared with those whose mothers were not exposed to watching television. Further studies are needed to provide the possible explanation for this seemingly counter-intuitive finding.

It is clear from literature [43, 44] that female households do not face the barrier of seeking permission from anybody before utilizing health services for their children and themselves, and would expect them to have higher odds of seeking treatment for children. However, this study noted that children whose household head was female were less likely to undergo diarrhoea treatment compared with those whose household head was male. This could plausibly due to health seeking going beyond just permission to include other socio economic factors such are financial challenges [45]. We, therefore, suggest that further studies be conducted to address this contradiction with available studies.

### Strengths and limitations

This study has some strengths and limitations. One of the strengths is the use of a relatively large sample size and representative datasets from 30 sub-Saharan African countries which make the findings from this study generalizable to other children of women in the countries considered in this study. Also, this study used multilevel modeling and this model accounted for the nested/ hierarchical nature of the datasets to provide reliable estimates. In terms of limitations, the study used cross-sectional data, which limits causal interpretations of the results. Moreover, since the study is self-reported, recall bias may exist and may lead to over or under reporting.

### Implications for public health, policy, and practice

There are several policy and practice implications from this study for promoting the utilization of ORT, zinc, and other treatments in the management of diarrhoea among under-five children in SSA. This study found a varied country-specific prevalence of the use of ORT, zinc, and other treatments in the management of diarrhoea among under-five children. The low prevalence of diarrhoea treatment among under-five children in SSA could scale up if the prevention and treatment of diarrhoea becomes a national priority among countries in SSA and these countries commit to the number of key actions outlined by the United Nations Children’s Fund and World Health Organization in 2009 [46]. Some of these key actions include mobilizing sufficient resources for diarrhoea control; expanding health services into communities and ensuring that diarrhoea prevention and treatment is central to the “revitalization” of community-based primary health care approaches. Also, considering the key predictors of diarrhoea treatment in this study, governments across SSA must focus on these predictors when implementing policies and public health intervention strategies to scale up diarrhoea treatment among under-five children in the sub-region. These efforts would further reduce under-five morbidity and mortalities related to diarrhoea in SSA.

## Conclusion

The study found that the prevalence of diarrhoea treatment among children in SSA was relatively low. Between-country variations in the prevalence of diarrhoea treatment was recorded. The sub-regional estimates of diarrhoea treatment and identified associated factors can support country-specific needs assessments targeted at improving policy makers’ understanding of within-country disparities. Planned interventions (e.g., provision of quality and affordable supply of ORS, zinc, and other treatments) should focus on scaling up diarrhoea treatment among under-five children in SSA, with much focus on the factors identified in this study.

## Abbreviations

aOR: Adjusted Odds Ratio
CI: Confidence Interval
CHW: Community Health Worker
DHS: Demographic and Health Survey
EAs: Enumeration Areas
ICC: Intra-Class Correlation
ORS: Oral Rehydration Salt
ORT: Oral Rehydration Therapy
PSU: Primary Sampling Unit
RHF: Recommended Homemade Fluid; SSA: sub-Saharan Africa
STROBE: Strengthening the Reporting of Observational Studies in Epidemiology

## References

[1] Koletzko S, Osterrieder S. Acute infectious diarrhea in children. Deutsches Ärzteblatt International. 2009; 106(33): 539. doi: 10.3238/arztebl.2009.0539 19738921 PMC2737434

[2] Reiner RC Jr, Graetz N, Casey DC, Troeger C, Garcia GM, Mosser JF, et al. Variation in childhood diarrheal morbidity and mortality in Africa, 2000–2015. New England Journal of Medicine. 2018; 379(12): 1128–1138. doi: 10.1056/NEJMoa1716766 30231224 PMC6078160

[3] Das JK, Salam RA, Bhutta ZA. Global burden of childhood diarrhea and interventions. Current Opinion in Infectious Diseases. 2014; 27(5): 451–458. doi: 10.1097/QCO.0000000000000096 25101554

[4] Naghavi M, Abajobir AA, Abbafati C, Abbas KM, Abd-Allah F, Abera SF, et al. Global, regional, and national age-sex specific mortality for 264 causes of death, 1980–2016: a systematic analysis for the Global Burden of Disease Study 2016. The Lancet. 2017; 390(10100): 1151–1210. 10.1016/S0140-6736(17)32152-9 PMC5605883 28919116

[5] Moore SR, Lima NL, Soares AM, Oriá RB, Pinkerton RC, Barrett LJ, et al. Prolonged episodes of acute diarrhea reduce growth and increase risk of persistent diarrhea in children. Gastroenterology. 2010; 139(4): 1156–1164. doi: 10.1053/j.gastro.2010.05.076 20638937 PMC2949449

[6] Sarkar R, Tate JE, Ajjampur SS, Kattula D, John J, Ward HD, et al. Burden of diarrhea, hospitalization and mortality due to cryptosporidial infections in Indian children. PLoS neglected tropical diseases. 2014; 8(7): e3042. doi: 10.1371/journal.pntd.0003042 25058664 PMC4109911

[7] MacDonald V, Banke K. Creating an Enabling Environment for Scaling up Diarrhoea Treatment with Zinc and ORS. European Journal of Nutrition & Food Safety. 2015; 638–639. doi: 10.9734/EJNFS/2015/21005

[8] Sreeramareddy CT, Low YP, Forsberg BC. Slow progress in diarrhea case management in low and middle income countries: evidence from cross-sectional national surveys, 1985–2012. BMC pediatrics. 2017; 17(1): 1–8. 10.1186/s12887-017-0836-6 28320354 PMC5360044

[9] Ugwu J, Ezeagu I, Ibegbu M. Awareness and practice of zinc therapy in diarrheal management among under-five caregivers in Enugu State, Nigeria. International Journal of Medicine and Health Development. 2019; 24(2): 63–9. doi: 10.4103/ijmh.IJMH_13_19

[10] Yeshaw Y, Worku MG, Tessema ZT, Teshale AB, Tesema GA. Zinc utilization and associated factors among under-five children with diarrhea in East Africa: A generalized linear mixed modeling. *PloS One*. 2020; 15(12): e0243245. doi: 10.1371/journal.pone.0243245 33264367 PMC7710063

[11] Kawakatsu Y, Tanaka J, Ogawa K, Ogendo K, Honda S. Community unit performance: factors associated with childhood diarrhea and appropriate treatment in Nyanza Province, Kenya. BMC Public Health. 2017; 17(1): 1–14. 10.1186/s12889-017-4107-0 28209194 PMC5314605

[12] Sood N, Wagner Z. Private sector provision of oral rehydration therapy for child diarrhea in sub-Saharan Africa. The American journal of tropical medicine and hygiene. 2014; 90(5): 939. doi: 10.4269/ajtmh.13-0279 24732456 PMC4015590

[13] Wagner Z, Asiimwe JB, Dow WH, Levine DI. The role of price and convenience in use of oral rehydration salts to treat child diarrhea: A cluster randomized trial in Uganda. PLoS Medicine. 2019; 16(1): e1002734. doi: 10.1371/journal.pmed.1002734 30677019 PMC6345441

[14] Ajayi DT, BO IT, Oke OA, Fabiyi GA. Determinants of Oral Rehydration Solution and Zinc Use Among Under-Five Children for The Management of Diarrhea in Abeokuta, Nigeria. Archives of Basic and Applied Medicine. 2019; 7: 35–9.

[15] Workie HM, Sharifabdilahi AS, Addis EM. Mothers’ knowledge, attitude and practice towards the prevention and home-based management of diarrheal disease among under-five children in Diredawa, Eastern Ethiopia, 2016: a cross-sectional study. BMC Pediatrics. 2018; 18(1): 1–9. 10.1186/s12887-018-1321-6 30453926 PMC6241041

[16] Gwarzo GD. Mothers’ awareness and use of zinc in under-five children with diarrhoea in North-Western Nigeria. Nigerian Journal of Paediatrics. 2018; 45(2): 81–85. 10.4314/njp.v45i2.2

[17] Larson CP, Saha UR, Nazrul H. Impact monitoring of the national scale up of zinc treatment for childhood diarrhea in Bangladesh: repeat ecologic surveys. PLoS medicine. 2009; 6(11): e1000175. doi: 10.1371/journal.pmed.1000175 19888335 PMC2765636

[18] Corsi DJ, Neuman M, Finlay JE, Subramanian S. Demographic and health surveys: a profile. International Journal of Epidemiology. 2012; 41(6): 1602–1613. doi: 10.1093/ije/dys184 23148108

[19] Von Elm E, Altman DG, Egger M, Pocock SJ, Gøtzsche PC, Vandenbroucke JP, et al. The Strengthening the Reporting of Observational Studies in Epidemiology (STROBE) Statement: guidelines for reporting observational studies. International Journal of Surgery. 2014; 12(12): 1495–1499. doi: 10.1016/j.ijsu.2014.07.013 25046131

[20] Wiens KE, Lindstedt PA, Blacker BF, Johnson KB, Baumann MM, Schaeffer LE, et al. Mapping geographical inequalities in oral rehydration therapy coverage in low-income and middle-income countries, 2000–17. The Lancet Global Health. 2020; 8(8): e1038–e1060. doi: 10.1016/S2214-109X(20)30230-8 32710861 PMC7388204

[21] Wilson SE, Morris SS, Gilbert SS, Mosites E, Hackleman R, Weum KL, et al. Scaling up access to oral rehydration solution for diarrhea: Learning from historical experience in low- and high-performing countries. Journal of Global Health. 2013; 3(1): 010404. doi: 10.7189/jogh.03.010404 23826508 PMC3700030

[22] Lenters LM, Das JK, Bhutta ZA. Systematic review of strategies to increase use of oral rehydration solution at the household level. BMC Public Health. 2013; 13 Suppl 3(Suppl 3): S28. doi: 10.1186/1471-2458-13-S3-S28 24564428 PMC3847633

[23] Das JK, Lassi ZS, Salam RA, Bhutta ZA. Effect of community based interventions on childhood diarrhea and pneumonia: uptake of treatment modalities and impact on mortality. BMC Public Health. 2013; 13 Suppl 3(Suppl 3): S29. doi: 10.1186/1471-2458-13-S3-S29 24564451 PMC3953053

[24] Lam F, Abdulwahab A, Houdek J, Adekeye O, Abubakar M, Akinjeji A, et al. Program evaluation of an ORS and zinc scale-up program in 8 Nigerian states. Journal of Global Health. 2019; 9(1): 010502. doi: 10.7189/jogh.09.010502 31073399 PMC6505637

[25] White EE, Downey J, Sathananthan V, Kanjee Z, Kenny A, Waters A, et al. A community health worker intervention to increase childhood disease treatment coverage in rural Liberia: A controlled before-and-after evaluation. American Journal of Public Health. 2018; 108(9): 1252–1259. doi: 10.2105/AJPH.2018.304555 30024811 PMC6085010

[26] Marbaniang SP. Women Care and Practices in the Management of Childhood Diarrhea in Northeast India. Child Care in Practice. 2020; 1–13. 10.1080/13575279.2020.1812534

[27] Agustina R, Sari TP, Satroamidjojo S, Bovee-Oudenhoven IM, Feskens EJ, Kok FJ. Association of food-hygiene practices and diarrhea prevalence among Indonesian young children from low socioeconomic urban areas. BMC public health. 2013; 13(1): 1–12. doi: 10.1186/1471-2458-13-977 24138899 PMC3813984

[28] Lamberti LM, Walker CLF, Noiman A, Victora C, Black RE. Breastfeeding and the risk for diarrhea morbidity and mortality. BMC public health. 2011; 11(3): 1–12. doi: 10.1186/1471-2458-11-S3-S15 21501432 PMC3231888

[29] Santos FS, Santos LHD, Saldan PC, Santos FCS, Leite AM, Mello DFD. Breastfeeding and acute diarrhea among children enrolled in the family health strategy. Texto & Contexto-Enfermagem. 2016; 25. 10.1590/0104-070720160000220015

[30] Tobin EA, Isah EC, Asogun DA. Care giver’s knowledge about childhood diarrheal management in a rural community in South-South Nigeria. International Journal of Community Research. 2014; 3(4): 93–99.

[31] Mekonnen M, Bekele K, Jemal K, Hailu D, Tesfa B, Mulatu T. Prevalence of Oral Rehydration Therapy Use During the Diarrheal Episode and Associated Factors Among Mothers of Under-Five Children Visiting Public Health Facilities in North Showa Zone, Oromia Region, Ethiopia. Patient preference and adherence. 2021; 15: 423. doi: 10.2147/PPA.S295428 33654387 PMC7910076

[32] Tsehay CT, Aschalew AY, Dellie E, Gebremedhin T. Feeding Practices and Associated Factors During Diarrheal Disease Among Children Aged Less Than Five Years: Evidence from the Ethiopian Demographic and Health Survey 2016. Pediatric Health, Medicine and Therapeutics. 2021; 12: 69. doi: 10.2147/PHMT.S289442 33633479 PMC7901551

[33] Adongo PB, Tapsoba P, Phillips JF, Tabong PTN, Stone A, Kuffour E, et al. The role of community-based health planning and services strategy in involving males in the provision of family planning services: a qualitative study in Southern Ghana. Reproductive health. 2013; 10(1): 1–15. doi: 10.1186/1742-4755-10-36 23890362 PMC3726500

[34] Story WT, Burgard SA, Lori JR, Taleb F, Ali NA, Hoque DE. Husbands’ involvement in delivery care utilization in rural Bangladesh: A qualitative study. BMC pregnancy and childbirth. 2012; 12(1): 1–12. doi: 10.1186/1471-2393-12-28 22494576 PMC3364886

[35] Akinyemi JO, Banda P, De Wet N, Akosile AE, Odimegwu CO. Household relationships and healthcare seeking behaviour for common childhood illnesses in sub-Saharan Africa: a cross-national mixed effects analysis. BMC health services research. 2019; 19(1): 1–11. 10.1186/s12913-019-4142-x 31088474 PMC6518738

[36] Titaley CR, Dibley MJ, Roberts CL. Factors associated with underutilization of antenatal care services in Indonesia: results of Indonesia Demographic and Health Survey 2002/2003 and 2007. BMC public health. 2010; 10(1): 1–10. doi: 10.1186/1471-2458-10-485 20712866 PMC2933719

[37] Guvuriro S, Booysen F. Economic bargaining power and financial decision-making among married and cohabitant women in South Africa. Development Southern Africa. 2019; 36(4): 504–518. 10.1080/0376835X.2019.1581599

[38] Alemayehu M, Meskele M. Health care decision making autonomy of women from rural districts of Southern Ethiopia: a community based cross-sectional study. International Journal of Women’s Health. 2017;9:213. doi: 10.2147/IJWH.S131139 28458582 PMC5402916

[39] Biswas RK, Rahman N, Islam H, Senserrick T, Bhowmik J. Exposure of mobile phones and mass media in maternal health services use in developing nations: evidence from urban health survey 2013 of Bangladesh. Contemporary South Asia. 2021; 29(3): 460–473. 10.1080/09584935.2020.1770698

[40] Menon P, Nguyen PH, Saha KK, Khaled A, Kennedy A, Tran LM, et al. Impacts on breastfeeding practices of at-scale strategies that combine intensive interpersonal counseling, mass media, and community mobilization: results of cluster-randomized program evaluations in Bangladesh and Viet Nam. PLoS medicine. 2016; 13(10): e1002159. doi: 10.1371/journal.pmed.1002159 27780198 PMC5079648

[41] Alam Z, Higuchi M, Sarker MAB, Hamajima N. Mass media exposure and childhood diarrhea: a secondary analysis of the 2011 Bangladesh demographic and health survey. Nagoya Journal of Medical Science. 2019; 81(1): 31. doi: 10.18999/nagjms.81.1.31 30962653 PMC6433623

[42] El-Khoury M, Banke K, Sloane P. Improved childhood diarrhea treatment practices in Ghana: a pre-post evaluation of a comprehensive private-sector program. Global Health: Science and Practice. 2016; 4(2): 264–275. 10.9745/GHSP-D-16-00021 PMC4982250 27353619

[43] Gupta AK, Borkotoky MK, Kumar A. Household headship and infant mortality in India: evaluating the determinants and differentials. International Journal of MCH and AIDS. 2015; 3(1): 44. 27621985 PMC4948170

[44] Gurmu E, Etana D. Household structure and children’s nutritional status in Ethiopia. Genus. 2013; 69(2): 113–130. https://www.jstor.org/stable/genus.69.2.113

[45] World Health Organization. Diarrhoea: why children are still dying and what can be done. 2009. Available at: https://apps.who.int/iris/bitstream/handle/10665/44174/9789241598415_eng.pdf. Accessed on 28th October, 2021.

[46] Amin R, Shah NM, Becker S. Socioeconomic factors differentiating maternal and child health-seeking behavior in rural Bangladesh: A cross-sectional analysis. International Journal for Equity in Health. 2010 Dec;9(1):1–1. doi: 10.1186/1475-9276-9-9 20361875 PMC2859349

